# Automated segmentation of macular edema for the diagnosis of ocular disease using deep learning method

**DOI:** 10.1038/s41598-021-92458-8

**Published:** 2021-06-28

**Authors:** Zhenhua Wang, Yuanfu Zhong, Mudi Yao, Yan Ma, Wenping Zhang, Chaopeng Li, Zhifu Tao, Qin Jiang, Biao Yan

**Affiliations:** 1 College of Information Science, Shanghai Ocean University, Shanghai, 201306 China; 2 The Affiliated Eye Hospital, Nanjing Medical University, Nanjing, 200025 China; 3 Department of Ophthalmology, The First People’s Hospital of Huai’an City, Huai’an, 223001 China; 4 Department of Ophthalmology, The Firstirst Affiliated Hospital of Soochow University, Suzhou, 215006 China; 5 Eye Institute, Eye and ENT Hospital, Shanghai Medical College, Fudan University, 83# Fen Yang Road, Shanghai, 200030 China

**Keywords:** Biotechnology, Computational biology and bioinformatics, Mathematics and computing

## Abstract

Macular edema is considered as a major cause of visual loss and blindness in patients with ocular fundus diseases. Optical coherence tomography (OCT) is a non-invasive imaging technique, which has been widely applied for diagnosing macular edema due to its non-invasive and high resolution properties. However, the practical applications remain challenges due to the distorted retinal morphology and blurred boundaries near macular edema. Herein, we developed a novel deep learning model for the segmentation of macular edema in OCT images based on DeepLab framework (OCT-DeepLab). In this model, we used atrous spatial pyramid pooling (ASPP) to detect macular edema at multiple features and used the fully connected conditional random field (CRF) to refine the boundary of macular edema. OCT-DeepLab model was compared against the traditional hand-crafted methods (C-V and SBG) and the end-to-end methods (FCN, PSPnet, and U-net) to estimate the segmentation performance. OCT-DeepLab showed great advantage over the hand-crafted methods (C-V and SBG) and end-to-end methods (FCN, PSPnet, and U-net) as shown by higher precision, sensitivity, specificity, and F1-score. The segmentation performance of OCT-DeepLab was comparable to that of manual label, with an average area under the curve (AUC) of 0.963, which was superior to other end-to-end methods (FCN, PSPnet, and U-net). Collectively, OCT-DeepLab model is suitable for the segmentation of macular edema and assist ophthalmologists in the management of ocular disease.

## Introduction

Macular edema is clinically defined as the accumulation of serous fluid within retina with increased central retinal thickness. It is the dominant sign of several ocular diseases including diabetic retinopathy, age-related macular degeneration, and retinal vein occlusion^1,2^. Clinical diagnosis, etiology identification, and treatment of macular edema have been greatly improved with the development of modern imaging technologies, especially optical coherence tomography (OCT). Fluid accumulation can be noninvasively observed and located in a clinical setting by OCT technology^3^.

The use of optical coherence tomography (OCT) enable the physicians to identify macular edema in its early or subtle manifestations, which can assist disease management and design of future trials. OCT is a non-invasive diagnosing tool, which can enabled fast, non-invasive, high-resolution visualization of ocular structure^4,5^. However, these are still several limitations existed in its application. Continuous hardware improvements have been achieved since OCT inception, but no significant progress has been made in the software analysis of OCT images^6^. Increasing number of patients required disease management based on OCT images in the clinical practices. However, the great number of OCT images and poor reproducibility between OCT assessors have often been reported.

Interpretation of OCT image is still a laborious, time-consuming, and challenging work for ophthalmologists. Currently, manual segmentation of macular edema by the highly trained physician is considered as the gold standard^7,8^. However, the potential fatigue of human experts lead to segmentation errors. Several computer-aided methods have been used in the segmentation of macular edema, including threshold-based, graph-based, active contours-based, and region-based approaches^9–12^. However, these methods were designed based on the hand-crafted features, which was highly dependent on the quality of OCT images and crafted based on domain knowledge.

Deep learning is a form of machine learning using the convolutional neural network, which has been used for healthcare and image analysis^13^. In deep learning, convolutional neural network (CNN) is a class of deep neural network, which is the most commonly applied to image analysis. CNN has been used for the segmentation of subretinal fluid, pigment epithelium detachment, and classification of retinal vasculature^14–16^. Due to the low computational efficiency and weak multi-scale feature extraction ability, improved CNNs were proposed including fully convolutional network (FCN), Pyramid scene parsing network (PSP-net) and U-net. FCN adds the full-connected layer in CNN network as convolution layer and connects the deconvolution layer to enhance the computational efficiency^17^. Pyramid scene parsing network (PSP-net) uses the pyramid level to separate the feature map into different sub-regions and form pooled representation for different locations to improve multi-scale feature extraction ability^18^. U-net is modified with up-sampling operators and a large number of feature channels to improve computational efficiency and multi-scale feature extraction ability^19^. Although these improved CNNs have better segmentation efficiency than convolutional neural network (CNN). However, there are still some challenges, such as fault-segmentation problem, over-segmentation problem, and multi-scale feature extraction problem caused by the limited depth of the convolutional network.

In this study, we proposed a deep learning method based on the DeepLab model for the segmentation of macular edema in OCT images (OCT-DeepLab). Atrous spatial pyramid pooling (ASPP) was used to segment the objects at multiple features to enhance the multi-scale feature extraction ability^20^. The fully connected conditional random field (FC-CRF) was then used to refine the boundary of macular edema to reduce the fault-segmentation and over-segmentation^21^. The segmentation performance of OCT-DeepLab was finally estimated by comparing against the hand-crafted methods (C-V and SBG) and the end-to-end methods (FCN, PSPnet, and U-net).

### Experimental principle of the propose method

**Figure 1:**
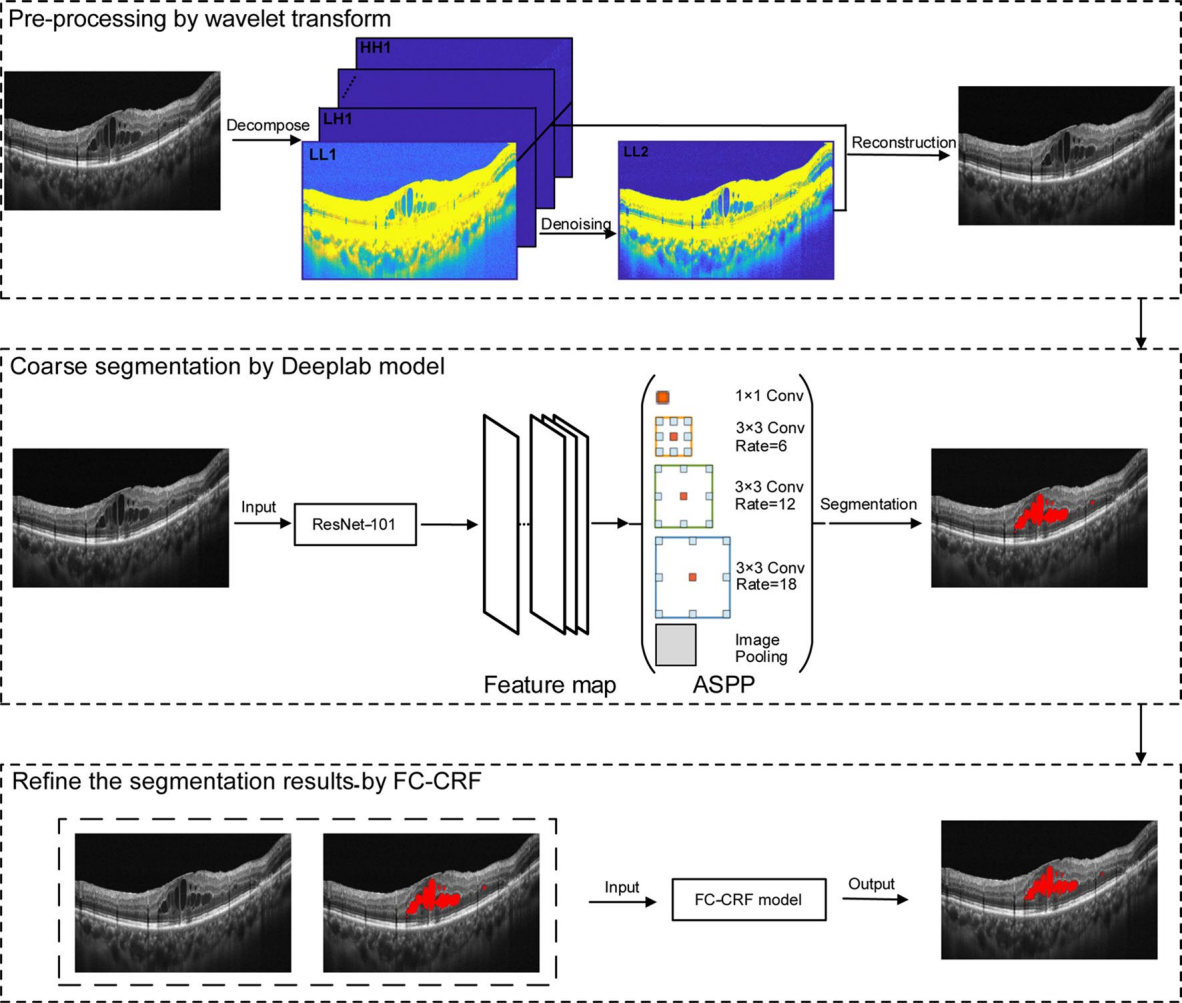
Flowchart of OCT-DeepLab method.

The flowchart of the proposed method, OCT-DeepLab, was shown in Fig. 1, including the pre-processing of OCT images by wavelet transform, the coarse segmentation of macular edema by DeepLab framework, and the boundary optimization by FC-CRF.

### Pre-processing of OCT images

**Figure 2:**
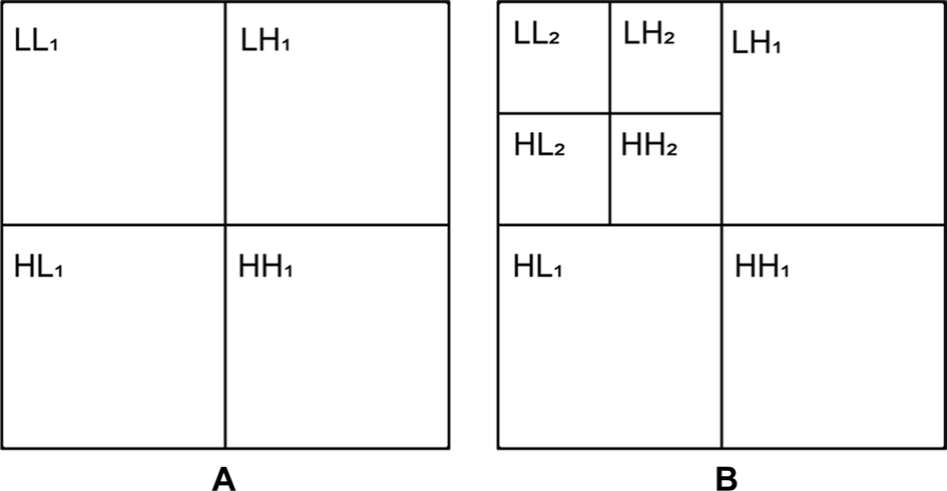
Flowchart of 2-level wavelet transform. (**A**) First level wavelet transform. (**B**) Second level wavelet transform.

Speckle noise can result in granular appearance, limit the contrast, and reduce the signal-to-noise ratio (SNR) of OCT images, which can pose great difficulties to identify the detailed features of OCT images^22,23^. In the pre-processing step, wavelet transform can reduce the speckle noises of OCT images^24^. OCT images are decomposed by two-level wavelet transform (Fig. 2). At the first level, OCT images ($LL_{0}$) is decomposed into a low frequency band ($LL_{1}$) and three high frequency band ($HH_{1}$, $LH_{1}$ and $HL_{1}$). At the second level, $LL_{i}$ is split into an approximation $LL_{{i + 1}}$ and three detail channels $LH_{{i + 1}}$ ,$HL_{{i + 1}}$ and $HH_{{i + 1}}$ for horizontally, vertically, and diagonally oriented details, respectively. The noise threshold (*NT*) of each low frequency band $LL_{{i + 1}}$ can differentiate between target signal and speckle noise.

*NT* is calculated by:

1 $$NT = \frac{{median(\left| {p_{{ij}} } \right|)}}{\alpha }$$

where $i$ and $j$ is the horizontal and vertical pixel coordinates of OCT images respectively;$p_{{ij}}$ is the pixel value.$\alpha$ is the hyperparameter, which can be used for rescaling denominator.

The process of reducing speckle noise is shown below:

2 $$p_{{ij}}^{{\prime }} = \left\{ {\begin{array}{*{20}l} {p_{{ij}} - NT,} \hfill & {p_{{ij}} > NT} \hfill \\ {0,} \hfill & {\left| {p_{{ij}} } \right| \le NT} \hfill \\ {p_{{ij}} + NT,} \hfill & {p_{{ij}} < - NT} \hfill \\ \end{array} } \right.$$

**Figure 3:**
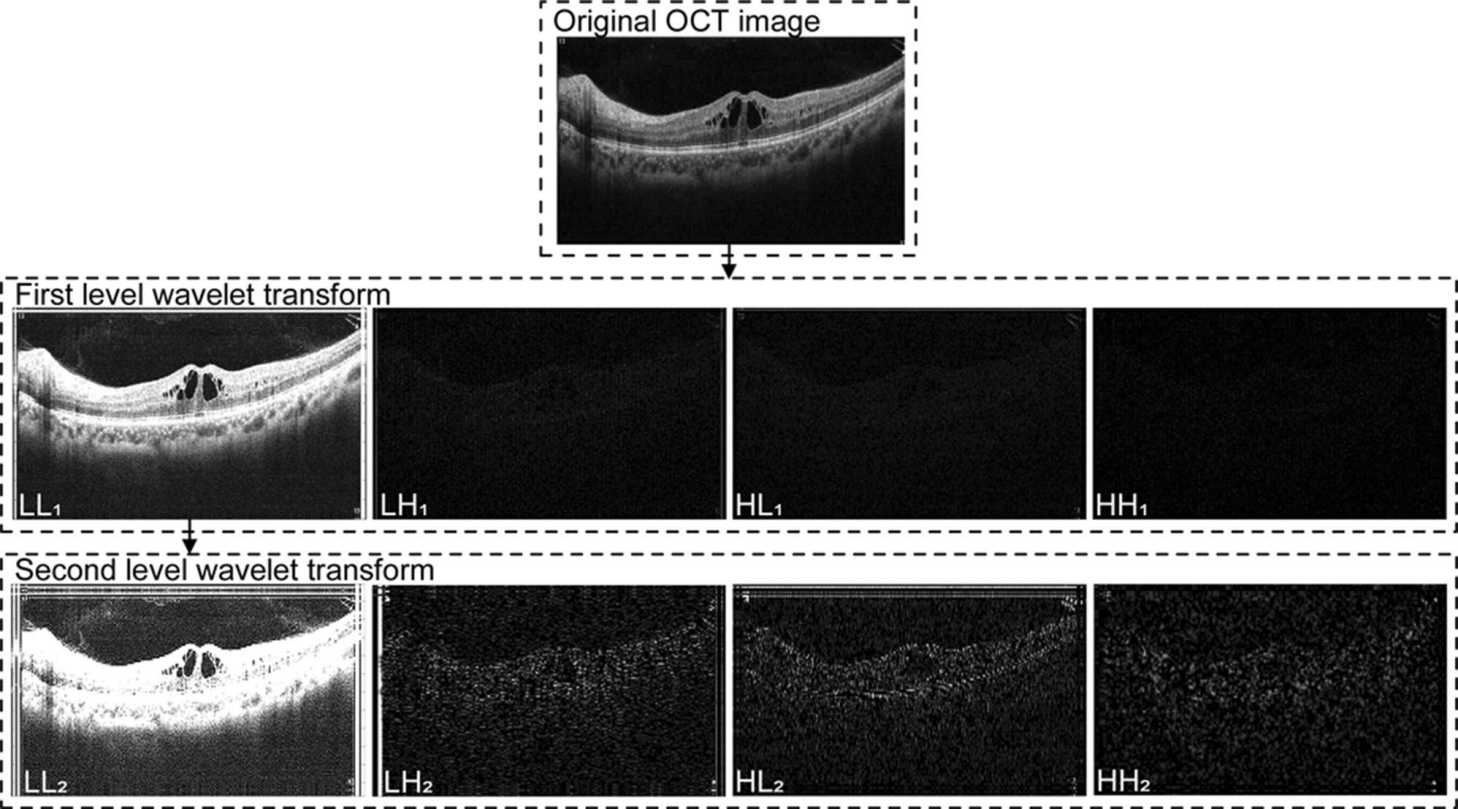
Decomposing process of OCT image by 2-level wavelet transform.

$p_{{ij}}^{{\prime }}$ is the pixel value after reducing noise. If $p_{{ij}} \le NT$, it denotes that the pixel is speckle noise and should be reduced. If $p_{{ij}} > NT$, it denotes that the pixel is the target signal and should be retained. OCT images are decomposed by 2-level wavelet transform (Fig. 3).

**Figure 4:**
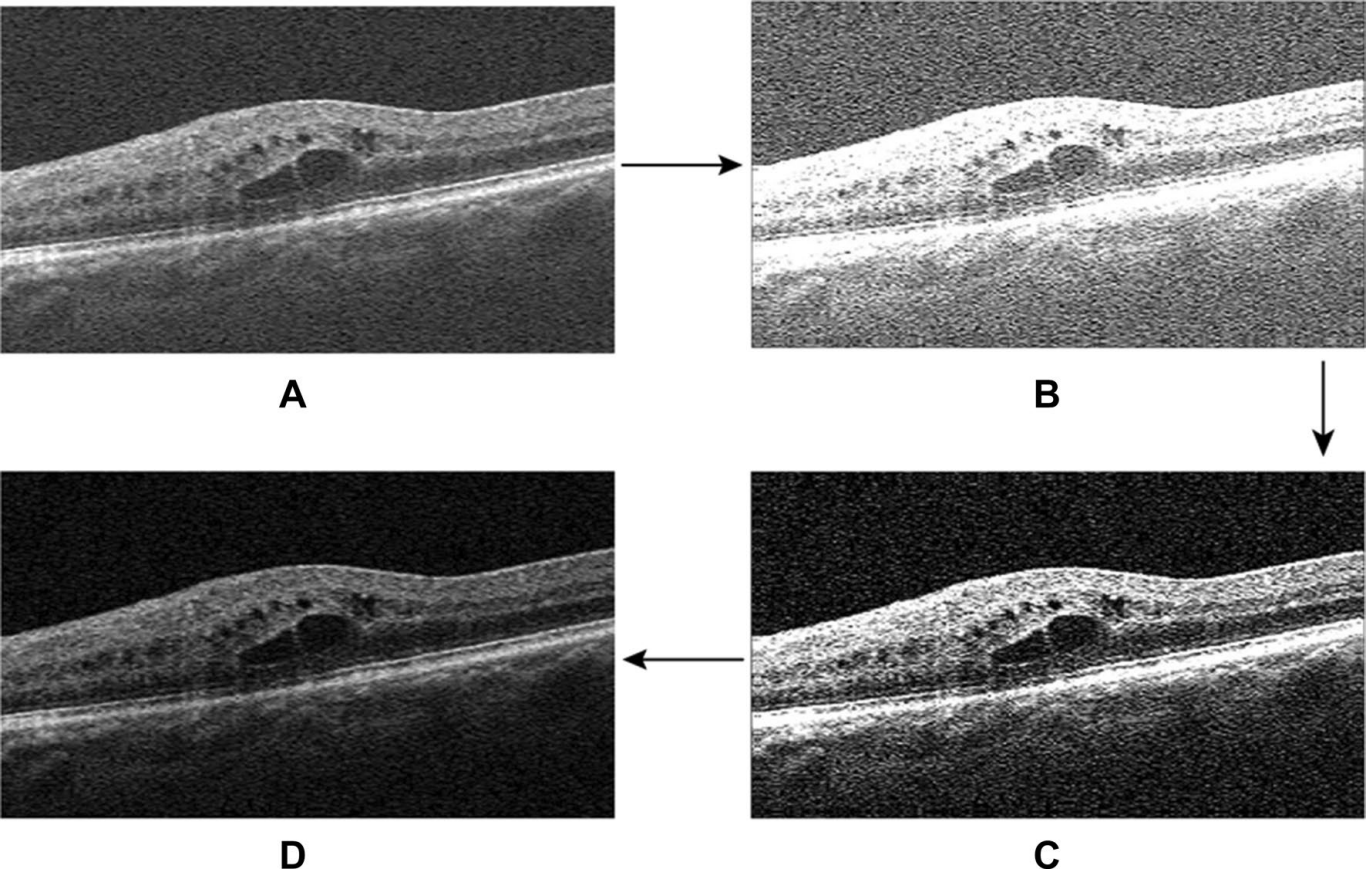
Denoising flowchart of OCT image. (**A**) Original OCT image. (**B**) Low-frequency band of OCT image. (**C**) Low-frequency band of OCT image by reducing speckle noises. (**D**) Reconstitution of OCT image.

The threshold of speckle noises (*NT*) for each OCT image is calculated by Eq. (1). Then, the speckle noises are reduced by Eq. (2). Figure 4 is the denoising flowchart of original image and the re-construction of new image.

### Coarse segmentation of macular edema in OCT images by Deeplab framework

At this step, macular edema is segmented by Deeplab framework. DeepLab is a deep learning model for image segmentation with deep convolutional nets, atrous spatial pyramid pooling (ASPP), and fully connected CRFs^20^. DeepLab uses the Resnet-101 with atrous convolutions as the main feature extractor and uses ASPP for extracting multiple scales features.

**Figure 5:**
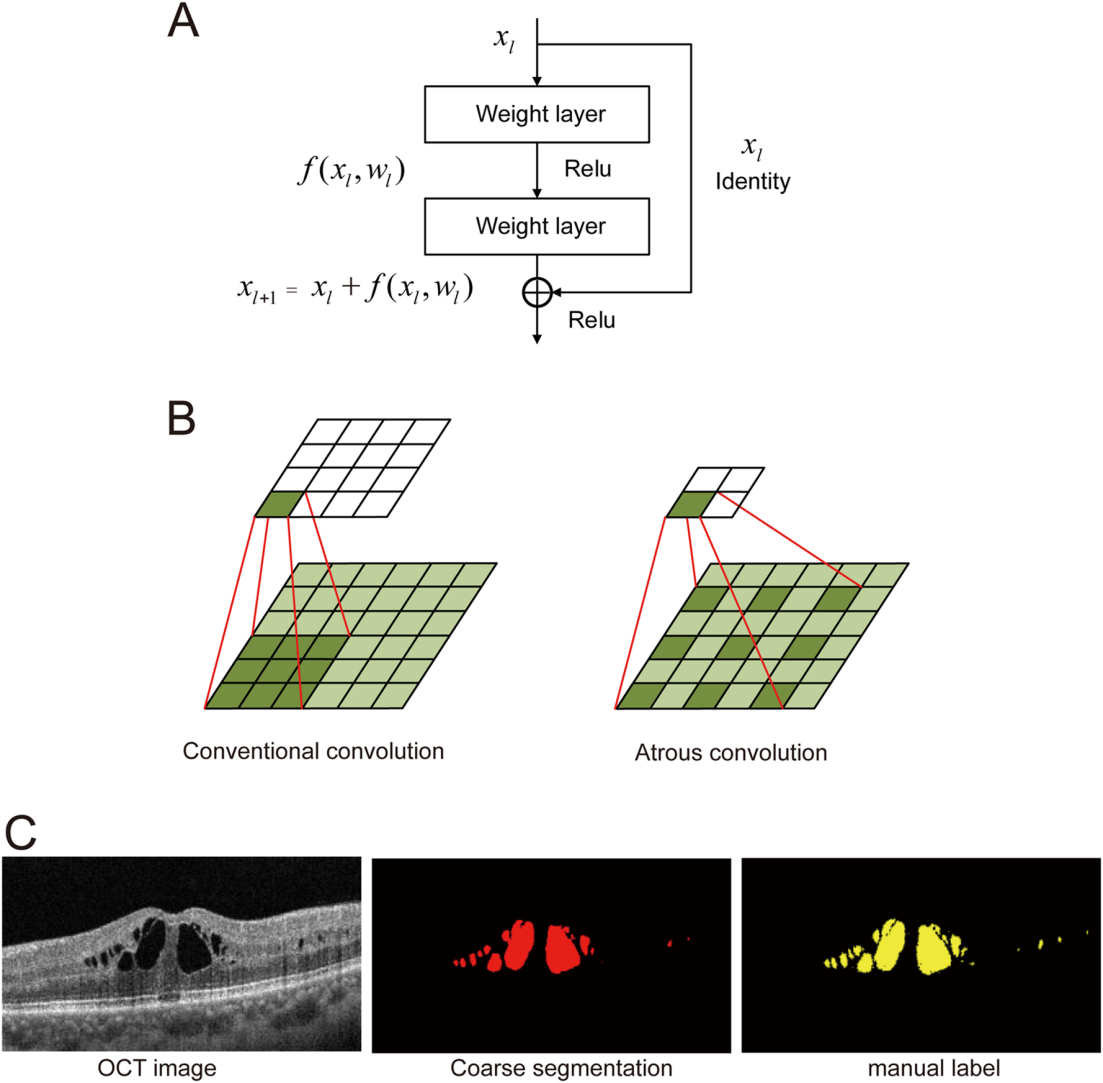
Principle for coarse segmentation of macular edema in OCT image. (**A**) Operating principle of residual learning block. (**B**) The workflow of conventional convolution and atrous convolution. Conventional convolution operation with 3 × 3 kernel size and 1 stride. Atrous convolution operation with 3 × 3 kernel size, 1 stride, and 2 rate. (**C**) Coarse segmentation of macular edema in OCT image.

Resnet-101 encoder addresses the degradation problem based on the residual learning block^25^, which is computed as shown below:

3 $$x_{{l + 1}} = x_{l} + f(x_{l} ,w_{l} )$$

where $f$ denotes the residual function; $x_{l}$ denotes the input feature to the $l$-th residual block; $w_{l}$ denotes a set of weights associated with $l$-th residual block. The operating principle of residual learning block is shown in Fig. 5A.

ASPP can extract the multiple scale features of OCT images by atrous convolution operation, which can enlarge the field of view of the kernel without suffering the increasing number of parameter’s problems^26^. Macular edema has different scales in OCT images. ASPP can account for different scales of macular edema which can improve the accuracy of segmentation.

Taking the re-constituted OCT images as the input dataset, the coarse segmentation of macular edema is conducted by OCT-Deeplab. Here, the learning rate of Deeplab neural network is set as 0.007, and then the value of learning rate is updated dynamically by the ‘poly’ optimization method. The value of momentum and weight decay is set as 0.9 and 5e−4, respectively^27^. Then, the coarse boundary of macular edema in OCT image is obtained (Fig. 5). Compared with the segmentation of macular edema by manual labels, the boundary of coarse segmentation of macular edema is smoother and fails to show the smaller scale of macular edema. Thus, the boundary of macular edema is required to be further optimized and refined.

### Refine the coarse segmentation result of macular edema by FC-CRF

At this step, the coarse boundary of macular edema is optimized by a probabilistic graphical model, FC-CRF. In the FC-CRF model, the segmentation of OCT image boundary is abstracted as an energy minimization problem.

The pixels of OCT image ($I$) are denoted as ${\text{X}} = \left\{ {X_{1} , \ldots ,X_{N} } \right\}$. The conditional random field $(I,X)$ is calculated by Gibbs distribution.

4 $$P\left( {X|I} \right) = \frac{1}{{Z(I)}}\exp \left( { - \sum\limits_{{c \in C\varsigma }} {\Phi _{c} (X_{c} |I)} } \right)$$

where $Z(I)$ is a normalization constant; $\varsigma$ is a graph associated with ${\text{I}}$; $c$ is a set of cliques $C_{\varsigma }$ in $\varsigma$, each inducing a potential $\Phi _{c}$^28^. The conditional probability of $X$ is caculated by Eq. (4). Gibbs energy function of $X$ is

5 $$E(X|I) = \sum\limits_{{c \in C\varsigma }} {\Phi _{c} (X_{c} |I)}$$

The maximum posteriori $X$ is obtained by minimizing the corresponding energy:

6 $$X^{*} = \arg \mathop {\min }\limits_{{X \in L}} E(X|I)$$

After minimizing $E(X|I)$, a binary segmentation of macular edema is obtained. Given a graph $\varsigma$ on $I$, its energy is obtained by summing its unary and pairwise potentials ($\psi _{u}$ and $\psi _{p}$, respectively):

7 $$E(X|I) = \sum\limits_{i} {\psi _{u} (X_{i} )} + \sum\limits_{{i < j}} {\psi _{p} (X_{i} ,X_{j} )}$$

where $i$ and $j$ range from 0 to *N*. The unary potential $\psi _{u} (X_{i} )$ defines a log-likelihood over the label assignment $X_{i}$. $\psi _{u} (X_{i} )$ is computed by a classifier.

**Figure 6:**
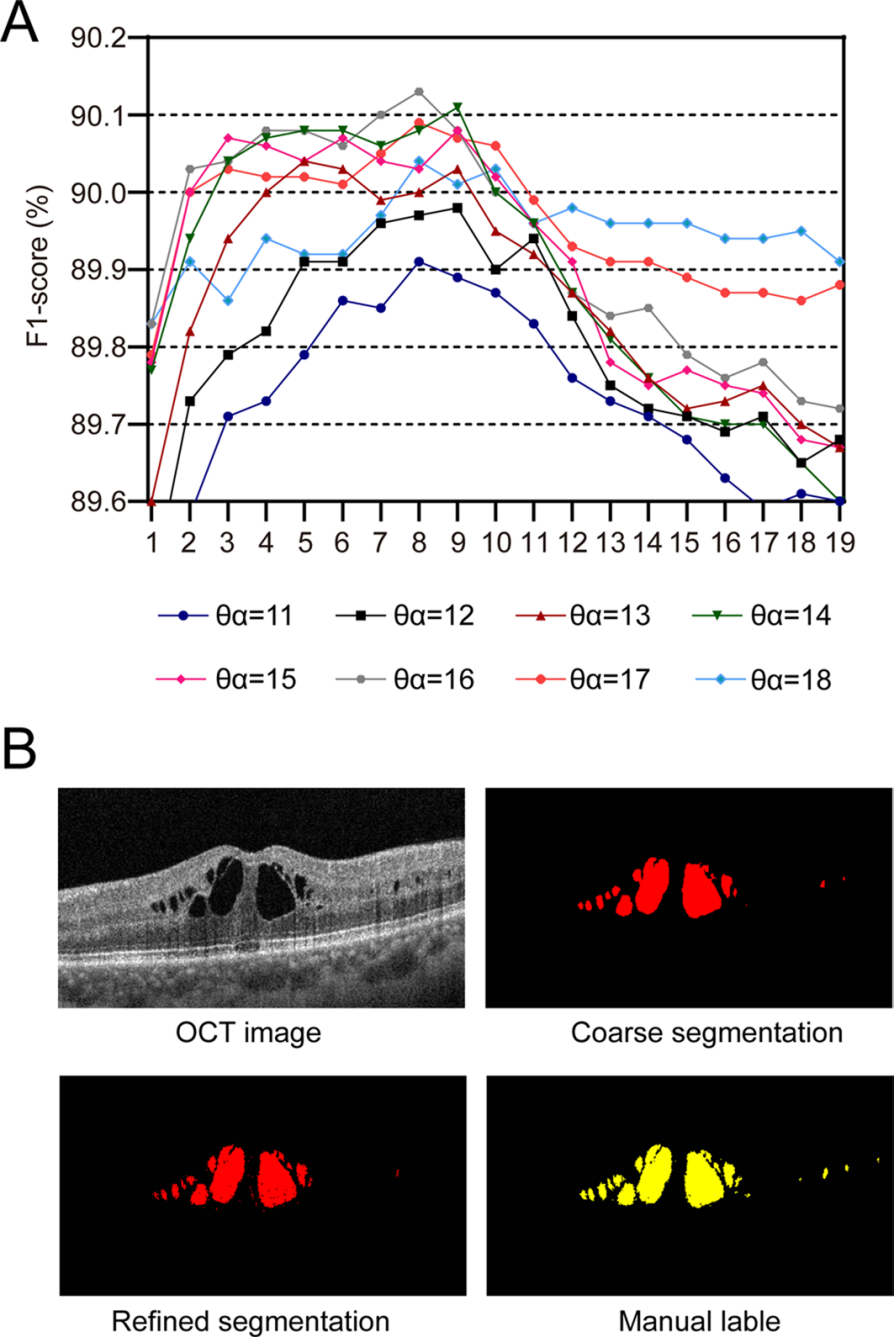
Refine the segmentation results of macular edema by FC-CRF. (**A**) Sensitivity test for FC-CRF model. (**B**) Refined segmentation result of macular edema in OCT images.

$\psi _{u} (X_{i} )$ is the coarse segmentation result of macular edema. The pairwise potentials is calculated as shown below:

8 $$\psi _{p} (X_{i} ,X_{j} ) = \mu (X_{i} ,X_{j} )\sum\limits_{{m = 1}}^{{\text{M}}} {\omega ^{{(m)}} k^{{(m)}} \left( {f_{i}^{{(m)}} ,f_{j}^{{(m)}} } \right)}$$

where $\mu (X_{i} ,X_{j} )$ is a label compatibility function; $\omega ^{{(m)}}$ is a linear combination weight; $k^{{(m)}} \left( {f_{i}^{{(m)}} ,f_{j}^{{(m)}} } \right)$ is a Gaussian kernels, which determines the similarity between connected pixels by means of $f^{{(m)}}$.

9 $$k^{{(m)}} \left( {f_{i}^{{(m)}} ,f_{j}^{{(m)}} } \right) = \exp \left( { - \frac{{\left| {p_{i} - p_{j} } \right|^{2} }}{{2\theta _{\alpha }^{2} }} - \frac{{\left| {f_{i}^{{(m)}} - f_{j}^{{(m)}} } \right|^{2} }}{{2\theta _{\beta }^{2} }}} \right)$$

were the vectors $f_{i}$ and $f_{j}$ are the feature vectors for pixel $i$ and $j$ in an arbitrary feature space; $p_{i}$ and $p_{j}$ are the coordinate vectors of pixel $i$ and $j$. $\theta _{\alpha }$ and $\theta _{\beta }$ are used to control the degrees of nearness and similarity between pixel $i$ and $j$. The proximity in distance ($\theta _{\alpha }$) and the similarity with the adjacent pixels ($\theta _{\beta }$) are the scale parameters of Gaussian kernel, which can refine the boundary of macular edema. Taking the parameter $\theta _{\alpha }$ as the fixed values, the changing curve of the parameter $\theta _{\beta }$ with respect to F1-score is shown in Fig. 6A, where $\theta _{\alpha }$ and $\theta _{\beta }$ range from 1 to 20, the step size is 1. When $\theta _{\alpha }$ = 16 and $\theta _{\beta }$ = 8, F1-score reach the peak value. FC-CRF can obtain the optimal result of segmentation. The refined result is shown in Fig. 6B. Compared with the coarse segmentation result, the refined segmentation results show more detail features of macular edema, which is close to the segmentation result of macular edema by manual labels.

## Results

### Compared the segmentation performance against with Deep lab with different setting

**Figure 7:**
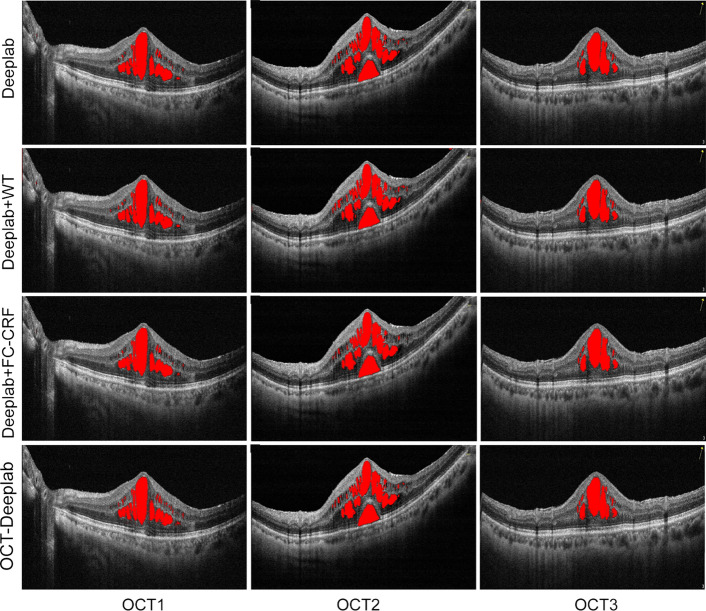
Different segmentation results of macular edema by Deeplab with different setting.

In order to evaluate the effect of wavelet transform and fully connected conditional random field on the segmentation performance, the proposed method against the Deeplab with different setting, including traditional Deeplab, Deeplab with wavelet transform (Deeplab + WT), Deeplab with fully connected conditional random field (Deeplab + FC-CRF).The different segmentation results of macular edema were shown in Fig. 7.

Table 1 showed the results of evaluation metrics for macular edema segmentation by Deeplab with different settings. Compared with the original Deeplab method, the Deeplab + WT method improved the scores of precision, specificity, F1-score of segmentation results, which were 94.73(2.92↑), 95.87(1.16↑), 92.82(0.95↑) respectively. And Deeplab + FC-CRF method improved the scores of precision, sensitivity, specificity and F1-score of segmentation results, which were 92.52(0.71↑), 96.56(4.25↑), 96.31(1.6↑), and 94.69(2.82↑) respectively. While our proposed OCT-DeepLab method achieved higher scores of precision, specificity, and F1-score than other methods, including Deeplab, Deeplab + WT and Deeplab + FC-CRF. While the segmentation results by OCT-DeepLab achieved higher scores of sensitivity than that of Deeplab and Deeplab + WT, and similar scores of sensitivity with that of Deeplab + FC-CRF.

**Table 1:** Performance comparison between Deeplab with different setting.

Model	Performance measures			
	Precision (%)	Sensitivity (%)	Specificity (%)	F1-score (%)
Deeplab	91.81 ± 0.64	92.31 ± 1.07	94.71 ± 1.29	91.87 ± 0.75
Deeplab + WT	94.73 ± 0.18	90.37 ± 1.88	95.87 ± 1.03	92.82 ± 1.12
Deeplab + FC-CRF	92.52 ± 0.23	96.56 ± 1.09	96.31 ± 0.92	94.69 ± 0.54
OCT-Deeplab	95.79 ± 0.11	95.16 ± 1.81	97.31 ± 1.32	95.43 ± 1.02

### Compared with traditional hand-crafted methods

**Figure 8:**
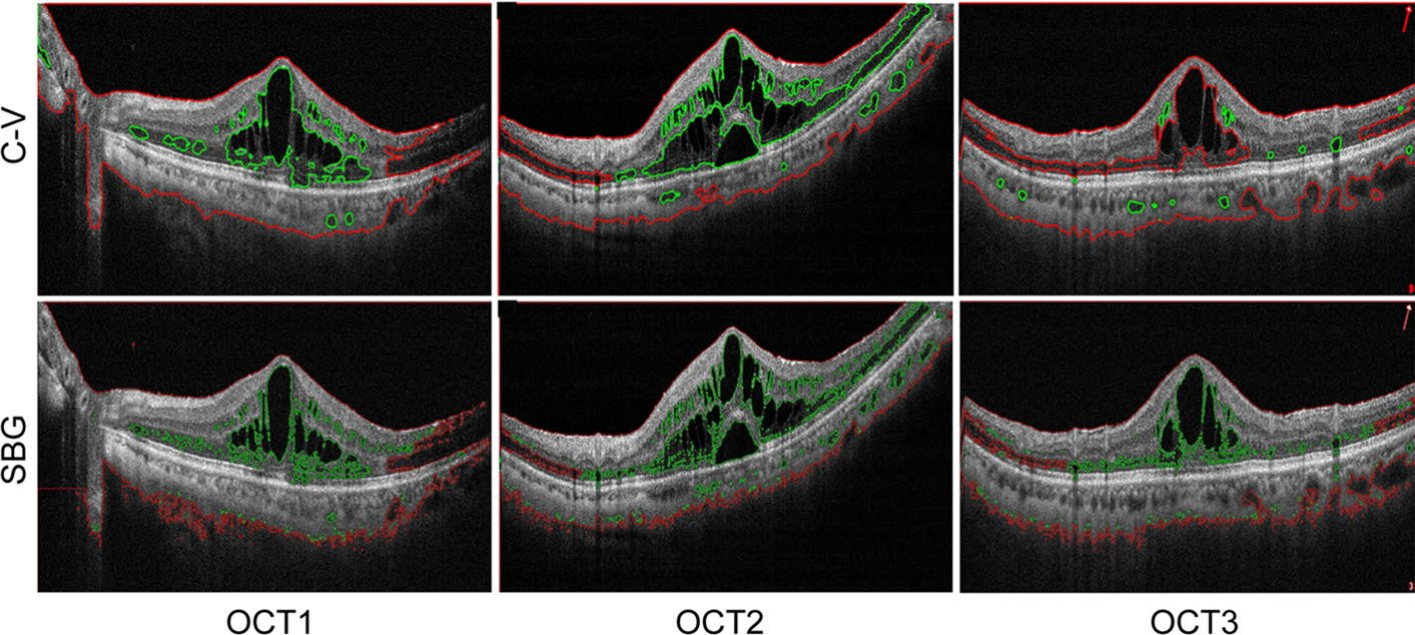
Segmentation results of macular edema by different methods. Red line is the initial contour curve of segmentation; Green line is the segmentation result of macular edema.

We compared our proposed method against other traditional hand-crafted methods, including C–V^29^ and SBG^9^, to evaluate the segmentation performance of macular edema. The segmentation results of macular edema were shown in Fig. 8.

As shown in Fig. 8, the red line is the initial contour curve of segmentation algorithm, while the green line is used to mark the segmented result of macular edema region. By the C-V model, a small part of macular edema region was identified. Several macular edema regions were omitted, especially for the precise segmentation of anomalous boundaries. By the SBG model, a part of retinal tissue was identified. OCT-DeepLab could accurately segment the region of macular edema. The segmentation results of OCT-DeepLab method showed great consistency with the segmentation results by manual labels. Table 2 showed the results of evaluation metrics. OCT-DeepLab achieved higher scores of precision, sensitivity, specificity, and F1-score than that of other traditional hand-crafted methods, including C–V and SBG.

**Table 2:** Performance comparison between different segmentation models.

Model	Performance measures			
	Precision (%)	Sensitivity (%)	Specificity (%)	F1-score (%)
C–V	72.18 ± 4.68	78.01 ± 4.36	83.37 ± 3.28	89.13 ± 5.13
SBG	81.65 ± 3.34	81.47 ± 1.79	88.13 ± 2.25	87.43 ± 3.72
OCT-Deeplab	95.79 ± 0.11	95.16 ± 1.81	97.31 ± 1.32	95.43 ± 1.02

Precision is a measure of relevance of results, and high precision attributes a method to yield accurate results. The advantage of OCT-DeepLab can be observed in the metric of precision, where OCT-DeepLab could achieve higher scores than C–V and SBG. The advantages of OCT-DeepLab can be also observed in the metrics of sensitivity, specificity, and F1-score. Sensitivity and specificity measure the proportion of relevant results. A high sensitivity means that the majority of all positive samples are truly detected. A high specificity means that the majority of all negative samples are truly detected. The sensitivity of OCT-DeepLab was greater than that of other methods. The higher score of sensitivity demonstrates that OCT-DeepLab can recognize more macular edema compared with the other two models. As for the specificity, OCT-DeepLab substantially exceeded C-V and SBG. F1-score is utilized to find the match between two similarities in the images. Its value also ranges between zero and one. The metric of F1-score in OCT-DeepLab was significantly greater than that in C-V and SBG, suggesting that the segmentation results of OCT-DeepLab is greatly consist with the segmentation results by manual labels.

### Comparison with other end-to-end methods

**Figure 9:**
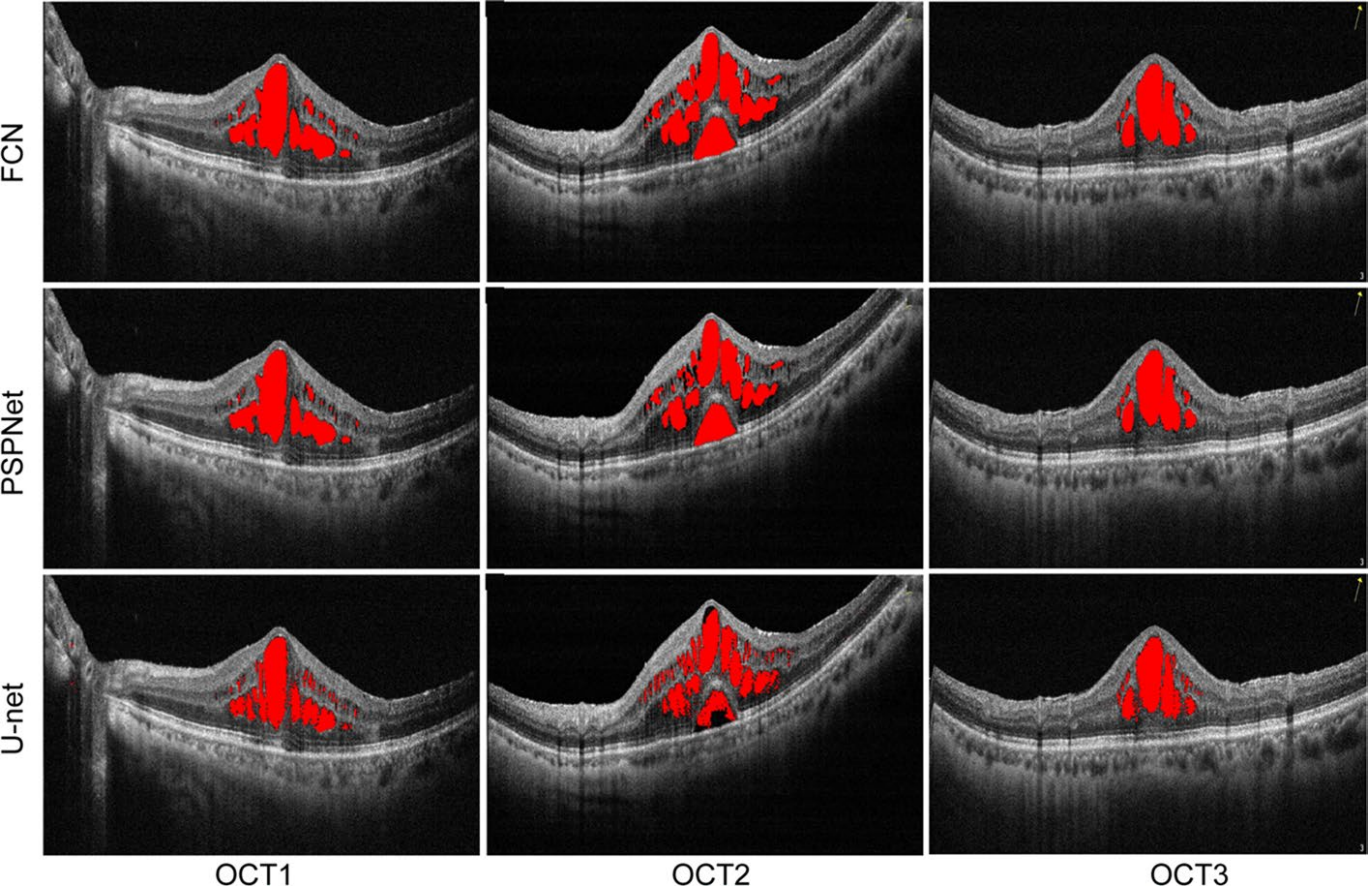
Segmentation results of macular edema by different methods.

In this section, we compared the proposed OCT-Deeplab method with the end-to-end methods, including FCN, PSPNet and U-net. The segmentation results of macular edema by different models were shown in Fig. 9.

In the FCN and PSPNet models, a part region of macular edema was misclassified and a small scale of macular edema were not correctly segmented. In the U-net model, the segmentation results of macular edema became clearer than FCN and PSPNet models, especially in small-scale macular edema regions. However, limited by the network structure of U-net, the input image size of U-net must be 32 or a multiple of 32. The segmentation result of our proposed is in agreement with the result of manual labels.

In order to reduce the influence of test data selection on experimental results, we use fivefolds cross-validation method to evaluate the performance of different methods. Table 3 shows the values of 4 different metrics for the segmentation of macular edema by different methods. OCT-DeepLab had better precision compared with FCN, PSPNet, or U-net. The precision of OCT-DeepLab was 95.79 which is over 84.30, 88.27, or 89.48 in FCN, PSPNet, or U-net by a large margin. The advantages of OCT-DeepLab were also observed in the metrics of sensitivity, specificity, and F1-score. The sensitivity of OCT-DeepLab was greater than that of other methods. The higher score of sensitivity demonstrates that OCT-DeepLab can recognize more macular edema compared with the other two models. As for the specificity, OCT-DeepLab substantially exceeded FCN, U-Net, and PSPNet. F1-score can determine the degree of similarities match between two images. OCT-DeepLab achieved higher scores than CN, PSPNet, or U-net, suggesting that OCT-Deeplab can obtain the closest results of macular edema segmentation to the results of manual labels.

**Table 3:** Performance comparison between different segmentation models.

Model	Performance measures			
	Precision (%)	Sensitivity (%)	Specificity (%)	F1-score (%)
FCN	84.30 ± 1.61	88.87 ± 1.81	93.35 ± 1.13	86.38 ± 0.96
PSPnet	88.27 ± 1.93	89.63 ± 0.52	93.50 ± 0.94	88.75 ± 0.98
U-net	89.48 ± 1.56	93.17 ± 2.01	94.53 ± 0.89	91.06 ± 0.53
OCT-Deeplab	95.79 ± 0.11	95.16 ± 1.81	97.31 ± 1.32	95.43 ± 1.02

**Figure 10:**
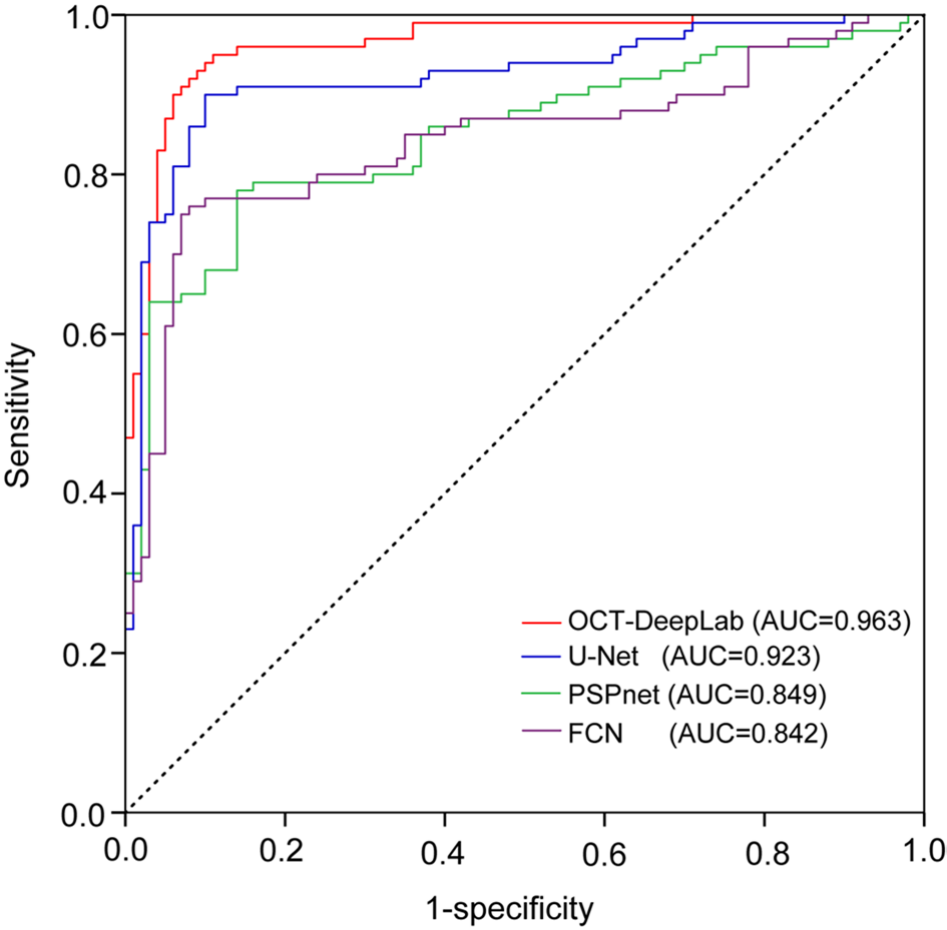
ROC curves from the segmentation of macular edema by different methods.

Receiver operating characteristics (ROC) analysis was then used to evaluate the performance for the segmentation of macular edema. The ROC curves for by different methods were shown in Fig. 10. Based on ROC curves, we computed the area under the curve (AUC). AUC represents the degree of separability between target regions and non-target regions. Higher the AUC, better the model is at distinguishing between macular edema and other retinal region. The performance of OCT-DeepLab was comparable to that of manual label, with an average area under the curve (AUC) of 0.963. Moreover, OCT-DeepLab had the greater value of AUC compared with that of other methods, suggesting that OCT-DeepLab shows better performance on distinguishing between macular edema and other retinal regions.

## Conclusion

A novel method based on DeepLab-based deep learning (OCT-DeepLab) was proposed to segment macular edema in OCT images, including: pre-processing of OCT images via speckle de-noising, coarse segmentation of macular edema based on atrous spatial pyramid pooling (ASPP), and refine the segmentation result of macular edema by FC-CRF. Compared with conventional CNNs or improved CNNs, OCT-DeepLab had better precision, sensitivity, specificity, and F1-score. OCT-DeepLab method can enhance the multi-scale feature extraction ability and reduce fault-segmentation and over-segmentation. This method will assist ophthalmologists for the detection of edema region and enhance the diagnosis efficiency.

In OCT-DeepLab method, atrous convolution is a powerful tool for the segmentation. Atrous convolution allows us to explicitly control the resolution and effectively enlarges the view field of filters to incorporate larger context without increasing the number of parameters or the amount of computation. Atrous spatial pyramid pooling (ASPP) can segment objects at multiple scales. ASPP probes a convolutional feature layer with filters at multiple sampling rates, thus capturing objects and image context at multiple scales. Moreover, the use of wavelet transform denoising further enhances the model's ability to segment small-scale lesions. In addition, the use of FC-CRF as a post-processing tool can refine the boundaries of macular edema and enhanced the accuracy of segmentation results.

In conclusion, we provide a deep learning method based on DeepLab framework to segment macular edema in OCT images. Due to its precision, reliability, and objectivity, it is a promising tool in the individual and the large-scale management of patients with ocular disease^30^. However, there are some limitations for this model. Due to the limited number of training samples in the given datasets, the segmentation results are comparatively not as high as the detection. As more data is accumulated in future, further improvements in the accuracy for macular edema segmentation in OCT images can be achieved.

## Methods

### Dataset

The large scale OCT image cohort was constructed with the collaboration of Eye Hospital (Nanjing Medical University), Suzhou First People's Hospital, and Huai'An First People's Hospital. The patients of diabetic macular edema who presented to the hospital between May 1, 2019 and June 30, 2020 were included. Exclusion criteria include recent pan-retinal photocoagulation, history of focal or grid laser, and other ophthalmologic diseases which may affect the accuracy of results. OCT images were centered on the macula with an axial resolution of 10 μm and a 24-bit depth and acquired in 2 s, covering a 4 × 4-mm area captured by Cirrus HD-OCT (Carl Zeiss Meditec, Inc., Dublin, CA, USA).Three medical students manually screened the data and removed unclassifiable images (i.e. signal-shielded and off-center). Three retinal specialists with more than 10-year clinical experience worked individually to label OCT images as ground truth. A senior expert was consulted in case of disagreement. The final dataset consists of 8676 volumetric OCT images from 6230 subjects. This study was approved by Ethics Committee of Eye Hospital (Nanjing Medical University) and followed the tenets of the Declaration of Helsinki. The written informed consent was obtained from all subjects.

### Evaluation experiments

To evaluate the performance on the segmentation of macular edema, three comparison experiments were conducted. In experiment 1, the proposed method was compared against Deeplab with different setting, including traditional Deeplab, Deeplab with wavelet transform (Deeplab + WT), Deeplab with fully connected conditional random field (Deeplab + FC-CRF). In experiment 2, the proposed method was compared against the traditional hand-crafted methods, including C-V and SBG. In experiment 3, the proposed method was compared against other end-to-end methods, including FCN, PSPNet, and U-net.

### Evaluation metrics

Four different metrics, including precision, sensitivity, specificity, and F1-score, were calculated to estimate the performance of segmentation as shown below:

10 $$Precision = \frac{{tp}}{{tp + fp}}$$

11 $$Sensitivity = \frac{{tp}}{{tp + fn}}$$

12 $$\begin{aligned} & Specificity = \frac{{tn}}{{fp + tn}} \\ & F1{\kern 1pt} - score = \frac{{2tp}}{{2tp + fp + fn}} \\ \end{aligned}$$

where $tp$, $fp$ and $fn$ denote the true positive region, false positive region and false negative region, respectively. F1-score is a balanced metric and determined by precision and sensitivity simultaneously.

Receiver operating characteristic (ROC) curves were then plotted to evaluate the overall segmentation performance. On the basis of ROC curves, we computed the area under the curve (AUC). AUC can be interpreted as the mean sensitivity value for all possible specificity values, or equivalently, as the mean specificity value for all possible sensitivity values. The possible AUC value ranges from 0.50 (discriminative performance equal to chance) to 1.00 (perfect discriminative performance).

### Implementation

In the evaluations of this paper, subsets of 3460, 1163 and 1053 images were randomly selected for training, validation and testing, respectively. The random sampling was at patient level so as to prevent leakage and biased estimation of testing performance. The validation stage is used to select and save the network parameters, and the test stage is used to test the generalization performance of the network. In this case, the proposed method can be compared with the state-of-theart methods. In terms of the hyper-parameters, the SGD optimizer is used to optimize the models with a learning rate that is initialized to 0.007 empirically. In the training stage, the number of epochs is 200 and the mini batch size is 8. All experiments were carried out on the Ubuntu 16.04 computer with 2 Intel Xeon CPUs, using a NVIDIA Tesla P100 16 GB GPU, and 256 GB of RAM. In addition, four different metrics are calculated to show the performance of the segmentation, which is a commonly-used metric in biomedical image segmentation.
